# The Effects of Monomer, Crosslinking Agent, and Filler Concentrations on the Viscoelastic and Swelling Properties of Poly(methacrylic acid) Hydrogels: A Comparison

**DOI:** 10.3390/ma14092305

**Published:** 2021-04-29

**Authors:** Claudia Mihaela Ninciuleanu, Raluca Ianchiş, Elvira Alexandrescu, Cătălin Ionuţ Mihăescu, Cristina Scomoroşcenco, Cristina Lavinia Nistor, Silviu Preda, Cristian Petcu, Mircea Teodorescu

**Affiliations:** 1 National Institute for Research and Development in Chemistry and Petrochemistry-ICECHIM, Spl. Independentei 202, 060021 Bucharest, Romania; claudia.ninciuleanu@yahoo.com (C.M.N.); ralumoc@yahoo.com (R.I.); elviraalexandrescu@yahoo.com (E.A.); mihaescu_catalin96@yahoo.com (C.I.M.); scomoroscencocristina@gmail.com (C.S.); lc_nistor@yahoo.com (C.L.N.); 2 Department of Bioresources and Polymer Science, Faculty of Applied Chemistry and Materials Science, Politehnica University of Bucharest, 1-7 Gh. Polizu Street, 011061 Bucharest, Romania; 3 Institute of Physical Chemistry “Ilie Murgulescu”, Romanian Academy, Spl. Independentei 202, 6th District, P.O. Box 194, 060021 Bucharest, Romania; predas01@yahoo.co.uk

**Keywords:** poly(methacrylic acid), composite hydrogels, viscoelastic properties, swelling degree, concentration effects

## Abstract

The present work aims at comparatively studying the effects of the concentrations of a monomer (10–30 wt% based on the whole hydrogel composition), crosslinking agent (1–3 mol% based on the monomer), and reinforcing agent (montmorillonite-MMT, 1–3 wt.% based on the whole hydrogel composition) on the swelling and viscoelastic properties of the crosslinked hydrogels prepared from methacrylic acid (MAA) and N,N′-methylenebisacrylamide (BIS) in the presence of K_2_S_2_O_8_ in aqueous solution. The viscoelastic measurements, carried out on the as-prepared hydrogels, showed that the monomer concentration had the largest impact, its three-time enhancement causing a 30-fold increase in the storage modulus, as compared with only a fivefold increase in the case of the crosslinking agent and 1.5-fold increase for MMT in response to a similar threefold concentration increase. Swelling studies, performed at three pH values, revealed that the water absorption of the hydrogels decreased with increasing concentration of both the monomer and crosslinking agent, with the amplitude of the effect of concentration modification being similar at pH 5.4 and 7.4 in both cases, but very different at pH 1.2. Further, it was shown that the increased pH differently influenced the swelling degree in the case of the hydrogel series in which the concentrations of the monomer and crosslinking agent were varied. In contrast to the effect of the monomer and crosslinking agent concentrations, the increase in the MMT amount in the hydrogel resulted in an increased swelling degree at pH 5.4 and 7.4, while at pH 1.2, a slight decrease in the water absorption was noticed. The hydrogel crosslinking density determinations revealed that this parameter was most affected by the increase in the monomer concentration.

## 1. Introduction

Hydrogels can be defined as hydrophilic polymeric networks formed from natural and/or synthetic polymers that are capable of absorbing and retaining a large amount of water or aqueous fluids without dissolving due to the crosslinked structure [1]. Over time, a wide variety of hydrogels have been obtained with applications in various fields such as medicine and pharmacy (drug delivery, tissue engineering, wound healing) [2], agriculture [3], electronic equipment industry [4], cosmetics industry [5], etc. The most important properties of hydrogels that must be taken into account in any application are the water content and the mechanical/viscoelastic properties, to which, depending on the use, others properties may be added, such as biocompatibility in the case of medical uses. However, the mechanical properties of hydrogels are generally low, which is a major disadvantage in many situations. The simplest methods of improving the mechanical properties of common hydrogels are based on adjusting the crosslinking degree by either changing the concentration of the monomer introduced in the synthesis process or by altering the crosslinking agent–monomer ratio, or adding a reinforcing agent; the water absorption of the hydrogels is simultaneously affected in all these cases [2,6,7].

Poly(methacrylic acid) (PMAA) hydrogels are pH-sensitive anionic materials with mucoadhesive properties. Because of this, they have found many applications in the drug-controlled release field, as they ensure protection to the drugs inside the stomach at acidic pH [8,9]. Garcia et al. [8] studied PMAA hydrogels for the controlled release of metoclopramide by varying the concentration of N, N′-methylenebisacrylamide (BIS) as a crosslinking agent. Following this study, the authors noted that the swelling degree decreased with an increase in the concentration of the crosslinking agent. A more detailed study of PMAA hydrogels was performed by Panis et al. [7], who showed that the xerogel density, the average molar mass between the crosslinking points, and the distance between the macromolecular chains increased, while the crosslinking degree decreased with increasing neutralization degree of the monomer. The methacrylic acid (MAA) concentration had a similar effect on the swelling degree, i.e., when a higher concentration of the monomer was used, the swelling degree decreased because more crosslinking points were formed in the network, leading to a matrix with a higher crosslinking density [7].

The literature also describes hydrogels based on PMAA that have been studied in combination with natural polymers (chitosan [10], starch [11], Salecan [12]), as well as copolymers of MAA with different monomers (acrylic acid [13], N-isopropylacrylamide [14], methacrylamide [15]) or hydrogels based on PMAA and poly(ethylene glycol) [16]. In the majority of cases, the hydrogels obtained were tested for the controlled release of various drugs such as theophylline, doxorubicin, and insulin. For example, Bajpai and Singh [15] investigated the swelling behavior of some poly (methacrylamide-co-MAA) hydrogels synthesized under various conditions and showed that by increasing the crosslinking agent amount, the swelling degree decreased. They also investigated the swelling behavior at different pH values (1–8) and noticed that the highest swelling degree was obtained for a pH value equal to 8, but a major change in volume of the hydrogels occurred starting with the 6–7 pH interval. Another important conclusion referred to the initial polymerization medium: when it was more diluted, the swelling degree increased because loop formation was favored against the creation of crosslinking points in the hydrogel network.

Mechanical properties are very important in the case of hydrogels used for controlled release, as well as for any other field. These properties can be improved by adding reinforcing agents such as clays (montmorillonite, laponite, kaolin etc.). Montmorillonite (MMT) is a natural clay that belongs to the smectite category, displaying the empirical formula Al_2_Si_4_O_10_ (OH)_2_ × yH_2_O. Its employment for the reinforcement of hydrogels is more advantageous economically because MMT is both readily available in nature and is environmentally friendly. Another important advantage of MMT is that it has a hydrophilic character and can be easily dispersed in the presence of hydrophilic polymers. Zhumagaliyeva et al. obtained bentonite-reinforced poly(acrylic acid)/PMAA composite hydrogels and studied the influence of several factors (temperature, crosslinking degree, clay content) on the swelling properties of hydrogels. They concluded that the swelling degree increased with pH and temperature and decreased with the concentration of the reinforcing agent [17]. Further, Junior et al. synthesized and characterized intercalated nanocomposite hydrogels based on PMAA and different concentrations of MMT (Cloisite Na, 5–20 wt.%) with potential applications in agriculture as controlled release systems [18] and studied the effect of clay concentration and environmental salinity on the swelling properties. Ianchis et al. studied PMAA composite hydrogels reinforced with commercial clay Cl93A [19], as well as the effect of several types of commercial clays, namely, ClNa (unmodified montmorillonite) and various organomodified types of MMT (Cl30B, Cl20A, and Cl15A) on the hydrogel network made up of PMAA and Salecan [20]. It was observed that ClNa was the most compatible clay with this type of network due to its hydrophilic character.

Crosslinked hydrogels based on PMAA or PMAA and MMT have potential uses in the controlled release of drugs [8,10,11,12] or agrochemicals [18] and wastewater purification [9].

The present work aimed to compare the effects of the monomer (MAA) and reinforcing agent (MMT) concentrations and crosslinking agent (BIS)/monomer (MAA) ratio on the viscoelastic properties and water absorption at various pH values of the crosslinked PMAA hydrogels. To the best of our knowledge, this is the first report of such a study. For a better comparison of the effect of the various components, the viscoelastic properties of hydrogels were investigated immediately after synthesis (“as-prepared hydrogels”), when their composition was identical to that of the precursor solution, while the water absorption/swelling degree was calculated in relation to the amount of polymer in the hydrogel only. We will show within this paper that, out of the three components mentioned which were employed within the usual concentration limits, the monomer concentration used in the synthesis process displhadayed the most pronounced influence on both the viscoelastic properties and crosslinking density of hydrogels. Further, the swelling degree of the PMAA hydrogels decreased with the concentration of the monomer and crosslinking agent and increased with the concentration of MMT; the resulting values depended on the pH of the medium, in agreement with the pH-sensitive nature of these types of hydrogels.

## 2. Materials and Methods

### 2.1. Materials

Unmodified montmorillonite (Cloisite Na, ClNa) provided by Southern Clay Products Inc. (Gonzales, TX, USA) was purified by magnetic stirring with distilled water (100 g/1 L distilled water) at room temperature for 24 h, followed by decantation for 24 h. The sedimented part was separated, and the supernatant was centrifuged at 900 rpm for 30 min; the collected sediment was dried by lyophilization and used in experiments. Methacrylic acid (MAA, 99% Janssen Chimica, Beerse, Belgium), N, N methylenebisacrylamide (BIS 99% Sigma Aldrich, Saint Louis, MS, USA), and ammonium persulfate (APS 98% Sigma Aldrich) were used as received. The solvent used in the synthesis of hydrogels was deionized water (DI, 18.2 MΩ resistivity). Swelling studies were performed in deionized water (pH 5.4), HCl solution (pH 1.2), and PBS (phosphate-buffered saline) solution (0.01 M, pH 7.4) obtained from tablets (Sigma) and deionized water according to the manufacturer’s instructions and adjusted as needed to obtain the desired pH.

### 2.2. Synthesis of Hydrogels

The clay was dispersed under magnetic stirring in the calculated amount of DI for 24 h, and the monomer and crosslinking agent were added, followed by the initiator. The polymerization mixture was then injected into a mold consisting of two glass plates separated by a 1-mm-thick Teflon gasket. The mold was sealed and placed in a thermostatted bath at 60 °C for 5 h. At the end of the polymerization process, the mold was cooled down, disassembled, and discs of 8 mm and 20 mm in diameter were cut from the hydrogel. The 8 mm discs were purified for seven days in DI, with the water being replaced every day, while the 20 mm discs were used immediately for rheological measurements. Table 1 displays the compositions of the investigated hydrogels. Three different series of hydrogels were investigated: series 1: variation in the monomer concentration (10, 20, and 30 wt% MAA based on the whole hydrogel composition); series 2: variation of the concentration of the crosslinking agent (1, 2, 3 mol% based on the monomer); series 3: variation of the filler (MMT) concentration (1, 2, 3 wt% based on the whole hydrogel composition).

To determine the monomer conversion, part of the hydrogel was dried first in air and then over anhydrous CaCl_2_ to a constant mass and weighed (Wo). The dry xerogel was then immersed for purification in DI, which was changed daily for seven days, and then dried again (Wext). The monomer conversion (C%) was calculated according to Equation (1):

C (%) = (Wext − W_MMT_)/(Wo − W_MMT_) × 100 (1)

where W_MMT_ is the amount of MMT contained in the material used to determine the conversion.

### 2.3. Determination of Swelling Degree and Crosslinking Density

To determine the swelling degree, the pre-weighed xerogels (W_X_) were immersed in 30 mL of swelling solution at 37 °C for 72 h or other various time intervals. The hydrogels were then removed from the solution, wiped superficially with filter paper, and weighed (W_H_). All the measurements were performed in duplicate. The swelling degree (SD) was calculated as the ratio of the amount of water absorbed to the mass of polymer in xerogel according to Equation (2):

SD (g/g) = (W_H_ − W_X_)/(W_X_ × (100 − %MMT/100)) (2)

where % MMT is the percentage of MMT in xerogel, calculated based on the amount of monomers and MMT introduced in the polymerization and the total monomer conversion.

The average molecular weight between crosslinks (${\bar{M}}_{c}$) and the crosslinking density ($\rho_{c}$) were determined in the case of the hydrogels swelled at pH 1.2 in order to avoid the influence of the base character of the aqueous MMT dispersion on the swelling degree of the composite hydrogels (see below). At pH 1.2, all the methacrylic acid units within the hydrogel were in the acid form, and therefore the hydrogels can be considered non-ionic. In the case of non-ionic hydrogels and neglecting the contribution of the chain-end defects, ${\bar{M}}_{c}$ can be calculated according to Equation (3) [21,22].

(3) $${\bar{M}}_{c}=-\frac{\left(1-\frac{2}{f}\right)V_{1}\upsilon_{2r}^{2/3}\upsilon_{2m}^{1/3}}{\bar{\upsilon }\left[\ln\left(1-\upsilon_{2m}\right)+\upsilon_{2m}+{\chi \upsilon }_{2m}^{2}\right]}$$

where f is the functionality of the crosslinking site (f = 4), V_1_ represents the molar volume of the solvent (for water V_1_ = 18 cm^3^/mol), $\upsilon_{2r}$ and $\upsilon_{2m}$ represent the polymer volume fraction of the as-prepared hydrogel and the hydrogel swelled at equilibrium, respectively, $\bar{\upsilon }$ is the specific volume of the polymer (0.775 cm^3^/g in the case of PMAA), while χ represents the Flory polymer—solvent interaction factor (0.48 for water—PMAA at 0% degree of ionization [23].

The polymer volume fractions ($\upsilon_{2r}$, $\upsilon_{2m}$) were calculated by using Equation (4) [24]

(4) $$\nu_{2r/m}={\left[1+\frac{\rho_{p}}{\rho_{s}}\times \left(\frac{W_{h}}{W_{x}}-1\right)\right]}^{-1}$$

where $\rho_{p}$ and $\rho_{s}$ are the polymer (1.29 g/cm^3^ for PMAA [25] and solvent densities (1.00 g/cm^3^ in the case of water), while W_h_ and W_x_ represent the mass of the hydrogel at equilibrium swelling ($\upsilon_{2m}$) or under as-prepared conditions ($\upsilon_{2r}$), and the mass of the corresponding xerogel, respectively.

The crosslinking density of the hydrogels was calculated according to Equation (5) [24].

(5) $$\rho_{c}=\frac{1}{\bar{\upsilon }\times {\bar{M}}_{c}}$$

### 2.4. Characterization of the Hydrogels

The rheological measurements were carried out at 25 °C on a Kinexus Pro instrument (Malvern Instruments, Malvern, UK, software 1.6) by using 20 mm parallel plates with roughened surface to avoid slippage. The normal force applied was 0.5 N. The amplitude sweep measurements were performed a 1 Hz constant frequency, while the frequency sweep experiments were carried out in the 0.1–10 Hz range, a deformation within the linear viscoelasticity range being applied.

The FTIR spectra were recorded on a Tensor 37 Bruker equipment (Woodstock, NY, USA) equipped with a Golden Gate ATR unit, by using ground xerogels.

X-ray diffraction (XRD) measurements were carried out on a Rigaku Ultima IV X-ray diffractometer (Tokyo, Japan) operated at 40 kV and 30 mA by employing xerogel powders.

The thermogravimetric analysis (TGA) of the xerogels was performed on a TA Q5000 IR (TA Instruments, New Castle, DE, USA) in a nitrogen atmosphere (40 mL/min), the samples being heated from room temperature to 700 °C with a 10 °C/min heating rate.

### 2.5. Statistical Analysis

The swelling experiments were performed in duplicate, and the results are presented as average values and standard deviation. Student’s *t*-test was used for assessment of statistical significance. Values were considered significant at *p* < 0.05.

## 3. Results and Discussion

The studied hydrogels were obtained by the radical copolymerization of MAA with BIS in aqueous solution, in the presence of APS as the initiator, by using different concentrations of the monomer, crosslinking agent, and MMT, within the usual concentration range employed for the synthesis of hydrogels. The standard hydrogel (SH), against which the concentration of the monomer and BIS was modified, while MMT was added in the case of composite hydrogels, contained 10 wt.% MAA and 2 mol% BIS. For a better comparison of the effect of the various components, the viscoelastic properties were measured on as-prepared hydrogels, whose composition was identical to the one of the precursor solution. Further, for a correct interpretation of the results, the water absorption/swelling degree was determined after the purification of the hydrogels and calculated in relation only to the amount of polymer in the hydrogel, excluding the amount of MMT incorporated in the case of composite hydrogels and taking into account the total monomer conversion. Because both the viscoelastic properties of hydrogels and their swelling degree depend on the monomer conversion, this was determined in each case, resulting very high conversions (90–98%), which allows the comparison of rheological and swelling measurements in all cases. To prove the incorporation of the reinforcing agent, the purified composite hydrogels were further characterized by FT-IR spectroscopy, XRD, and TGA analyses.

### 3.1. Influence of the Monomer Concentration

The change in the monomer concentration in the initial mixture influences the crosslinking degree [7,8,26] and the proportion of chain entanglements [26] within the hydrogels, and therefore their viscoelastic properties and swelling degree.

The effect of the monomer concentration was studied for three different values, namely, 10, 20, 30 wt% based on the whole reaction mass by keeping constant the crosslinking agent (BIS, 2 mol% to MAA) and the initiator (KPS 1 mol% to MAA) concentrations.

#### 3.1.1. Viscoelastic Properties

**Figure 1:**
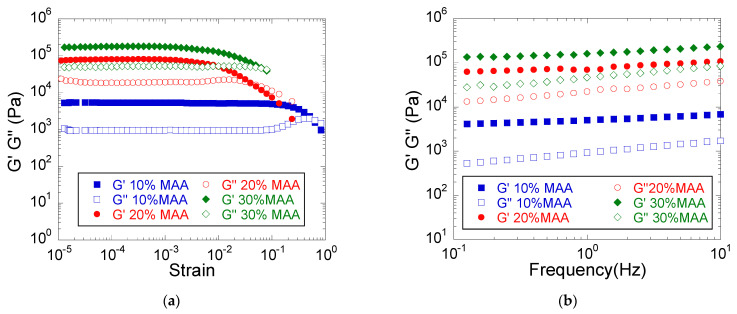
The influence of the monomer concentration on the viscoelastic properties of PMAA hydrogels (**a**) Amplitude sweep measurements (**b**) Frequency sweep measurements. Hydrogel composition: MAA, 2 mol% BIS based on MAA, 0% MMT.

The obtained hydrogels in their as-prepared state were characterized by both amplitude sweep and frequency sweep measurements. The amplitude sweep measurements (Figure 1a) showed that the linear viscoelasticity region (LVER) was wider at lower monomer concentrations, indicating a higher elasticity of the hydrogel at lower monomer concentrations, while the frequency sweep measurements (Figure 1b) demonstrated the crosslinked character of the hydrogels as indicated by the storage modulus (G′) being larger than the loss modulus (G″) over the entire frequency range investigated [27]. The values of the two viscoelastic moduli increased with the monomer concentration (Figure 1b, Table 2), showing that a more rigid hydrogel was developed in the case of higher amounts of the monomer, which can be explained by a higher polymer content of the hydrogel, i.e., a lower swelling degree [22], a higher crosslinking density (Table 2), and a larger proportion of entangled polymer chains. The progressively more rigid hydrogels with a denser network, formed with increasing monomer concentration, displayed a 10 times increase in the G′ value at 1 Hz when doubling the monomer concentration, and a 30 times increase in G′ when the monomer concentration was tripled (Table 2). Further, the increasing dependence of G′ and G″ on frequency indicated the presence of a network with large meshes in the case of all hydrogels due to the relatively low crosslinking agent/MAA mole ratio [27]. These results are in agreement with those of Mithra et al. [28], who investigated the viscoelastic properties of some sodium acrylate-based hydrogels with variable monomer contents. They similarly concluded that hydrogels with higher mechanical properties are obtained at higher monomer concentrations.

#### 3.1.2. Swelling Degree

**Figure 2:**
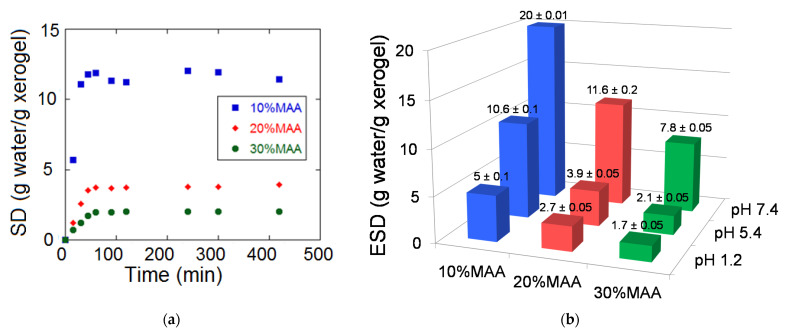
The influence of the monomer concentration on the swelling properties of hydrogels (**a**) Swelling curves at pH 5.4; (**b**) The effect of the monomer concentration on the swelling degree (72 h) at different pH values (1.2; 5.4; 7.4). The results express average values ± standard errors. Hydrogel composition: MAA, 2 mol% BIS based on MAA, 0% MMT.

In the case of environmentally sensitive hydrogels, the swelling degree may be influenced by the medium in which the swelling takes place. PMAA hydrogels belong to the pH sensitive hydrogel category due to the presence of acidic groups within their molecules. When the pH value of the swelling medium is higher than pKa, ionization of the acid groups in the hydrogel takes place, leading to the increase in the swelling degree [11]. The equilibrium swelling degree (ESD) of the hydrogels with different MAA contents was determined under various pH conditions (1.2; 5.4; 7.4) by SD measurements of the swelling degree as a function of time at 37 °C (Figure 2). The SD-time plots at pH 5.4 showed that swelling was fast, the equilibrium value being reached after less than 60 min (Figure 2a). However, the 10 wt.% monomer hydrogels were fragile and difficult to handle, and as a consequence, we decided to compare the swelling degree of the samples at 72 h (Figure 2b), a time interval sufficient to reach ESD according to the experiment mentioned above.

As we expected based on the pH-sensitive character of the hydrogels, regardless of the monomer concentration in the synthesis step, ESD increased with increasing pH, with the highest swelling degree being obtained at pH 7.4 (Figure 2b, Supplementary Material Figure S1a). The higher the pH of the medium, the higher the proportion of ionized COOH groups in the hydrogel, leading to higher electrostatic repulsions and an increase in the osmotic pressure in the hydrogel, both of them causing a higher water absorption at equilibrium. The increase in pH from 1.2 to 7.4 led to a significant increase in ESD for all three types of hydrogels (*p* < 0.05), namely, approximately four times in the case of the hydrogel synthesized at 10% MAA, 4.3 times for the one with 20 wt% MAA, and 4.6 times in the case of the 30 wt.% MAA hydrogel. Regardless of the pH value of the medium, the swelling degree increased with the decrease in the monomer concentration, in agreement with previous reports [7,15,21,25,26]. This can be explained by the decrease in the crosslinking density (Table 2), as a consequence of both a lower proportion of chain entanglements at lower monomer concentrations (Figure 2b) and the covalent crosslinking bridges being partially replaced by loops, resulting in intramolecular cyclization [7,15,21,25]. Tripling of the monomer concentration during synthesis led to a decrease in ESD of about 2.9 times at pH 1.2, five times at pH 5.4, and 2.6 times at pH 7.4. Therefore, it can be stated that the three types of hydrogels do not behave very differently from this point of view either (Supplementary Material Figure S2).

The crosslinking density, determined in the case of hydrogels that swelled at pH 1.2 (Table 2), displayed a strong increase with monomer concentration, because of the reasons shown above. In addition, the low value of ${\bar{M}}_{c}$ seems to indicate the formation of supplementary bridges through hydrophobic interactions among PMAA chains. The calculations showed that a 3-fold increase in the monomer concentration led to a 4.5-fold enhancement of the crosslinking density at this pH value.

### 3.2. Influence of the Crosslinking Agent Concentration

The concentration of the crosslinking agent in the hydrogel network is an important parameter to investigate because it directly affects the swelling degree and therefore the viscoelastic properties of the hydrogel. In this study, the effect of three different concentrations of the crosslinking agent was investigated, namely, 1, 2 and 3 mol% relative to the monomer, with the concentration of the monomer being 10 wt% based on the whole hydrogel composition.

#### 3.2.1. Viscoelastic Properties

The viscoelastic properties of hydrogels depend on their swelling degree [23] and therefore, to study the intrinsic effect of crosslinking agent concentration, measurements were made immediately after the synthesis of hydrogels, when the swelling degree was practically the same in all cases, dictated by the initial composition of the hydrogel.

**Figure 3:**
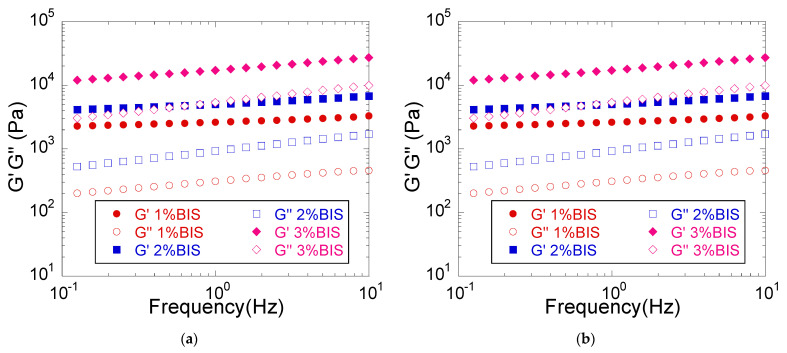
The influence of the crosslinking agent concentration on the viscoelastic properties of PMAA hydrogels (**a**) Amplitude sweep measurements (**b**) Frequency sweep measurements. Hydrogel composition: BIS, 10% MAA based on the whole hydrogel composition, 0% MMT.

The use of a higher concentration of crosslinking agent in the synthesis process leads to hydrogels with a higher density of crosslinking bridges, and therefore, more crosslinked structures are obtained that have higher mechanical properties. Indeed, the amplitude sweep and frequency sweep rheological measurements (Figure 3) showed that the hydrogels synthesized at a higher concentration of BIS were more rigid, displaying higher G′ values, in agreement with the expected formation of a more crosslinked network. Furthermore, the viscoelastic properties remained constant over a larger deformation range when the BIS concentration was lower, indicating a higher elasticity of the less crosslinked hydrogels (Figure 3a). Further, the frequency sweep measurements (Figure 3b) highlighted G′ > G″ over the entire frequency range studied, thus confirming the crosslinked character of the hydrogels [25]. The difference between G′ and G″ increased with decreasing frequency, indicating the presence of a wide-mesh network [22], in agreement with the BIS concentration used. Table 3 shows the values of the parameters G′ and G″ at 1 Hz frequency for the three crosslinking agent concentrations employed (1 mole%, 2 mol% and 3 mol% relative to the monomer), and it can be noticed that the doubling of the BIS concentration (2 mol% vs. 1 mole%) led to a doubling of G′, while tripling the crosslinking agent concentration (3 mol% vs. 1 mole%) caused a G′ increase of about fivefold. By comparison, the increase in the monomer concentration led to a much greater increase in G′ (Table 2), i.e., a 2-fold enhancement of the monomer concentration led to a 10-fold increase in G′, while tripling the monomer concentration resulted in an approximately 30-fold increase in G′, an effect which can be ascribed to both the increase in the crosslinking degree and the higher polymer concentration in the swollen hydrogel, as mentioned above [29,30].

#### 3.2.2. Swelling Degree

**Figure 4:**
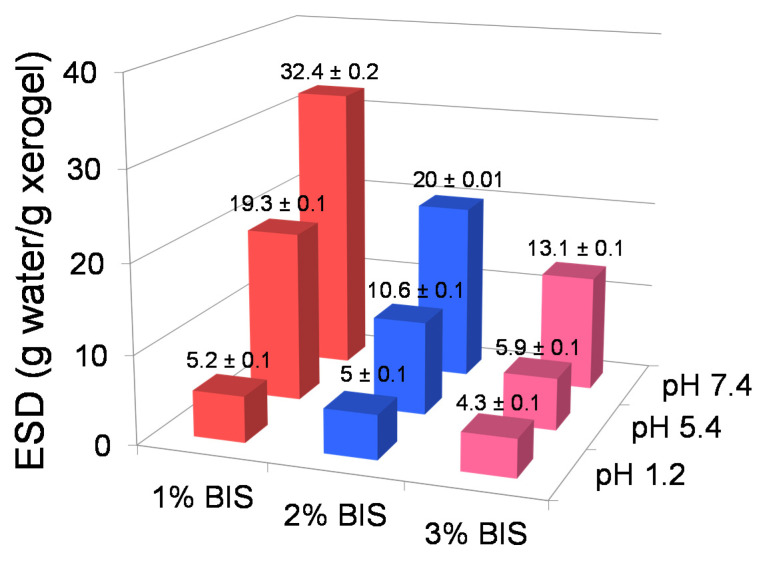
The influence of the crosslinking agent concentration on the swelling degree (72 h) at different pH values. The results are the average values ± standard errors. Hydrogel composition: BIS, 10% MAA based on the whole hydrogel composition, 0% MMT.

The adjustment of the crosslinking agent concentration is one of the most important methods of controlling the crosslinking density of a hydrogel and, as a consequence, of the ability to swell in water (Supplementary Material Figure S1a,b). Indeed, our experiments showed that by increasing the concentration of BIS, the swelling degree of the hydrogel decreased, regardless of the pH of the medium (Figure 4), due to a higher crosslinking density (Table 3). Tripling the BIS concentration resulted in a reduction in ESD of only 1.2 times at pH 1.2, as compared to approximately 3.3 times and 2.5 times at pH 5.4 and 7.4, respectively. In comparison with the hydrogels synthesized at various monomer concentrations, the effect of increasing BIS concentration on ESD had a relatively similar amplitude at pH 5.4 and 7.4, but the behavior was very different at acidic pH, when tripling the BIS concentration led to an insignificant decrease in ESD, as compared to a decrease of almost 3-fold in the previous case. This dissimilarity at pH 1.2 is the consequence of the large difference between the influence of the two parameters upon the crosslinking density: a 3-fold increase in the monomer concentration led to a 4.5 increase in $\rho_{c}$ as compared with only a 1.5-fold increase for the same increase in the crosslinking agent concentration.

Regarding the effect of the pH of the medium, in this case as well, the pH increase led to an increase in ESD as expected; the reason for this effect is explained above. It is worth noting, however, that the two series of hydrogels behaved differently from this of point view as well. Namely, while in the case of hydrogels obtained at different monomer concentrations, the ratio R = (ESD at pH 7.4)/(ESD at pH 1.2) practically did not depend on the monomer concentration at synthesis (R ≈ 4), in the case of the crosslinking agent concentration modification, R significantly decreased with the increase in the amount of BIS from about 6 for the hydrogel with 1 mol% BIS relative to the monomer, to 4 and about 3 in the case of hydrogels with 2 mol% BIS and 3 mol% BIS, respectively (*p* < 0.05). We do not have an explanation for this phenomenon at this time.

### 3.3. Influence of the Reinforcing Agent

This chapter describes the influence of the reinforcing agent concentration, which can affect the viscoelastic and swelling behaviors of the hydrogel together with monomer and crosslinking agent concentrations. Hydrogels with 10 wt.% monomer in the precursor solution were analyzed, with the concentration of the crosslinking agent being 2 mol% relative to the monomer. The concentrations of the investigated reinforcing agent were 1, 2, 3 wt% based on the whole mass of hydrogel, i.e., 9.65, 19.3, 28.95 wt% in comparison to the amount of monomers (Table 1). The composite hydrogels obtained were structurally characterized by FT-IR, TGA, and XRD analyses to highlight the presence of the reinforcing agent, and the rheological properties and water absorption in solutions with various pH values were determined. We performed SEM analyses for a representative sample (H2%MMT) (Supplementary Material Figure S3).

#### 3.3.1. FT-IR Analyses

**Figure 5:**
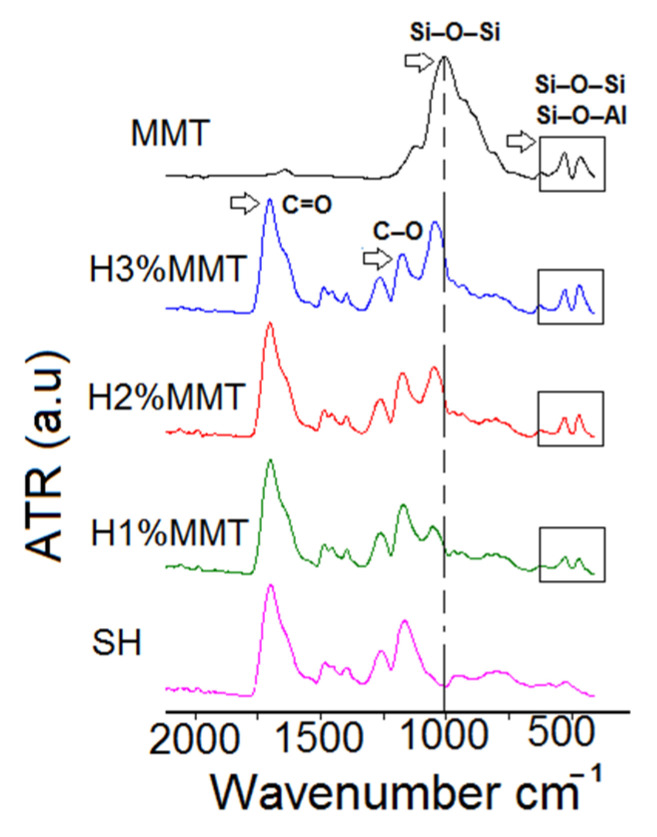
FT-IR spectra of the composite xerogels investigated and MMT. SH: 10%MAA, 2 mol%BIS based on MAA, 0%MMT.

FT-IR spectroscopy was used to prove the clay inclusion within the PMAA matrix and to detect whether interactions between components occurred. The FT-IR spectra of the composite xerogels displayed the characteristic peaks of the stretching vibrations of the Si–O–Si and Si–O–Al groups in MMT, thus confirming its presence in the material (Figure 5). We also noticed the increase in the intensity of the MMT characteristic peak in accordance with the amount of reinforcing agent introduced in the synthesis. In comparison to neat MMT, the characteristic peak of the clay at 999 cm^−1^ shifted to higher wavenumbers in the composite xerogels, indicating the presence of interactions between the clay layers and the PMAA network (Table 4). Further, the values of the peaks characteristic of the carboxylic group bonds (C=O, C–O) underwent shifts to higher wavenumbers in the case of the composite xerogels obtained, which reinforces the above statement.

#### 3.3.2. XRD Analyses

**Figure 6:**
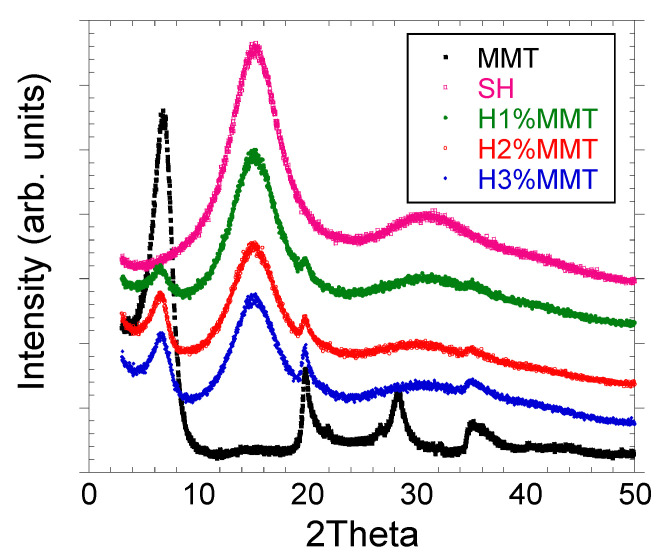
The diffractograms of MMT and the xerogels investigated. SH: 10%MAA, 2 mol%BIS based on MAA, 0%MMT.

The X-ray diffractograms (XRD) obtained for the studied composites and the pure MMT are displayed in Figure 6. By monitoring the position (2θ), shape, and intensity of the clay characteristic diffraction line in the composite structure, the intercalation/exfoliation phenomenon can be highlighted. In the case of SH, a peak characteristic of PMAA (2θ = 15°) was observed, which suggests the crystallinity of the polymer used in the synthesis of the hydrogel [19]. Once the MMT content of the xerogel increased, the intensity of this peak decreased, indicating the decrease in the crystallinity degree [31]. The analyses showed that in all cases, the peaks shifted to lower values (Table 5), which means that the d-spacing increased, suggesting that the clay layers were intercalated between the polymer chains.

#### 3.3.3. Thermogravimetric Analyses

**Figure 7:**
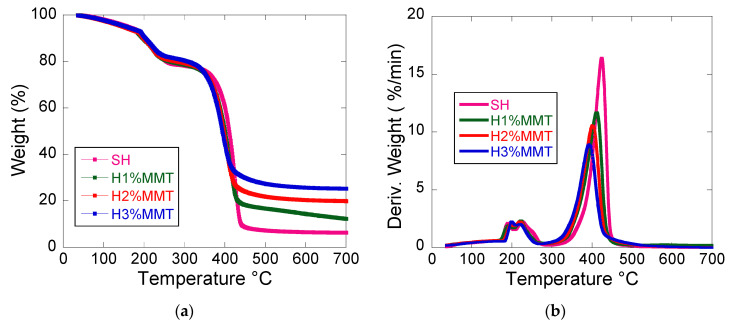
TGA (**a**) and TDG (**b**) curves of the obtained composite hydrogels. SH: 10%MAA, 2 mol%BIS based on MAA, 0%MMT.

The thermogravimetric analysis revealed the presence of three decomposition stages in the case of the nanocomposite hydrogels (Table 6, Figure 7). The first stage of decomposition that took place up to 120 °C was insignificant and can be attributed to the volatilization of the network water [12] still existing in the hydrogels.

In the second stage (120–300 °C), xerogel dehydration occurred by inter- and intramolecular elimination of water between COOH groups with the formation of anhydride cycles, as well as decarboxylation of COOH groups and CO_2_ elimination [27]. The last stage (300–700 °C) is ascribed to the total decomposition of the PMAA chains [28]. Total loss of mass was reduced in the case of composite hydrogels due to the presence of MMT that does not degrade at these temperatures. The residue value obtained by TGA analyses is often employed to demonstrate the presence and/or quantify the amount of inorganic agents used to reinforce polymers [29]. All MMT-containing samples showed a higher residue than the control sample at 700 °C, which increased with increasing MMT amount in the sample (Table 6). The residue amount was in good agreement with the initial percentage of MMT in the hydrogels (Table 1). The data in Table 6 also show that when the MMT content increased, T_2_ decreased. This phenomenon may be explained by a lower degree of crystallinity of the polymer at higher MMT concentrations (see XRD analyses), leading to a smaller decomposition temperature of the PMAA chains.

#### 3.3.4. Viscoelastic Properties

**Figure 8:**
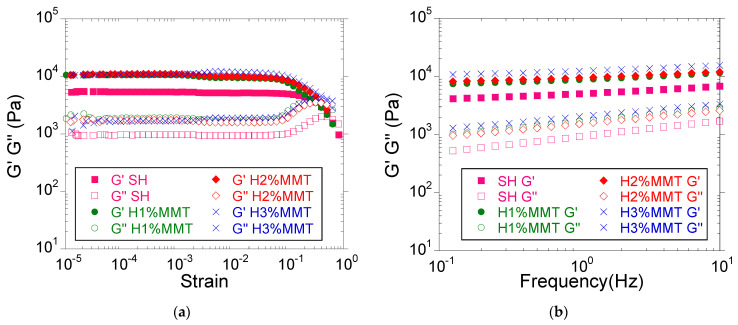
The influence of the MMT concentration on the viscoelastic properties of PMAA hydrogels (**a**) Amplitude sweep measurements (**b**) Frequency sweep measurements. SH: 10%MAA, 2 mol%BIS based on MAA, 0%MMT.

As in the case of the unreinforced hydrogels, the viscoelastic properties were investigated immediately after synthesis. Amplitude sweep measurements (Figure 8a) showed that even in the case of composite hydrogels, the viscoelastic properties were kept constant over a wide range of deformation (10^−5^–10^−1^), and therefore no appreciable decrease in elasticity (narrowing of LVER) with increasing amount of MMT was noticed. The frequency sweep measurements (Figure 8b, Table 7) showed that G′ was higher than G″ over the whole frequency range investigated, thus demonstrating the crosslinked character of the hydrogels [32,33,34].

**Figure 9:**
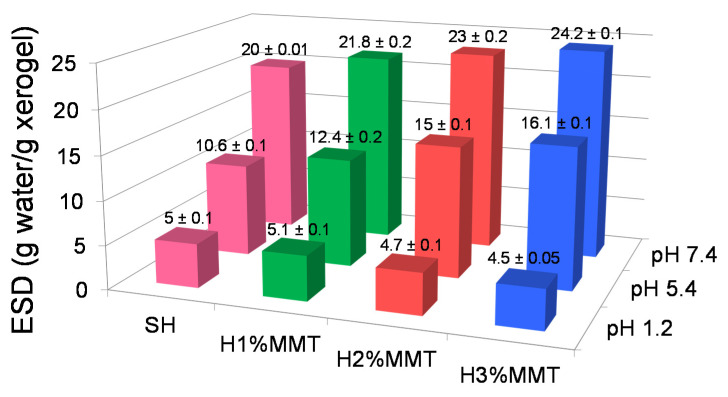
The influence of the reinforcing agent on the swelling degree (72 h) at different pH values. Results express average values ± standard errors. SH: 10%MAA, 2 mol%BIS based on MAA, 0%MMT.

The introduction of MMT into hydrogels led to an improvement of their mechanical properties, a fact demonstrated by the increase in the values of the viscoelastic modules with increasing clay concentration. For a 10 wt.% initial monomer concentration, G′ was approximately two times higher when 3 wt% MMT was used as compared to the unreinforced hydrogel (Figure 9). However, tripling the amount of the reinforcing agent in the hydrogel (from 1 wt.% to 3 wt.%) led to a relatively small increase in G′of about 1.5 times (from 8.8 × 10^3^ Pa to 12.4 × 10^3^ Pa, Table 7), much lower in comparison to the increase in G′ when the monomer concentration (≈30 times, Table 2) or crosslinking agent (≈six times, Table 3) tripled, which seems to indicate that the existing interaction between the clay and the network was weak in the presence of water.

#### 3.3.5. Swelling Degree

The influence of the amount of MMT on the swelling degree of the composite hydrogels was studied at pH 1.2, 5.4, and 7.4 (Figure 9). The results showed that the swelling degree significantly increased with increasing pH, as expected (*p* < 0.05), in agreement with the pH-sensitive character of these PMAA hydrogels (Supplementary Material Figure S1c). However, unlike the effect of the monomer and crosslinking agent concentrations, when larger amounts of these components led to a swelling decrease due to the formation of a more crosslinked network (Supplementary material Figure S2), increasing the concentration of MMT at pH 5.4 and 7.4 resulted in enhanced water absorption. This can be explained by the fact that MMT had very little influence on the crosslinking degree of the hydrogel (Table 7), but instead, because of its basic character (a 2 wt% MMT aqueous dispersion displayed a pH of 8.9), MMT led to the formation of carboxylate groups in the hydrogel during the synthesis stage, the proportion of which was higher for higher MMT concentrations. As a result, in ionizing media (pH 5.4 and 7.4), these already formed anionic charges added to the newly formed ones because of the environment, leading to a differentiation of hydrogels in terms of total charge. The higher the amount of MMT in the hydrogel, the higher the number of COO^-^ groups, leading to a higher swelling degree. It is worth noting that the ionization of COOH groups with the formation of negatively charged polymer chains begins at a pH higher than 5 [11]. The intrinsic effect of MMT on the swelling degree could be observed at pH 1.2, when, due to the strongly acidic medium, all carboxylate groups were transformed into carboxyl groups, and therefore the effect of MMT basicity was canceled [35]. The results showed that, at strongly acidic pH, the increase in MMT concentration led to a slight decrease in ESD (1.1 times for a 3-fold increase in MMT concentration—Figure 9), as a consequence of the enhanced crosslinking density (Table 7), similar to the effect of increasing BIS concentration under the same environmental conditions. This seems to indicate a slight crosslinking effect of MMT, which can be seen only at low swelling degrees.

SEM analyses performed for a representative sample (H2%MMT) swollen at three different pH values revealed that the hydrogels present large interconnected pores which change their size depending on the swelling medium (Supplementary Material Figure S3).

## 4. Conclusions

**Figure 10:**
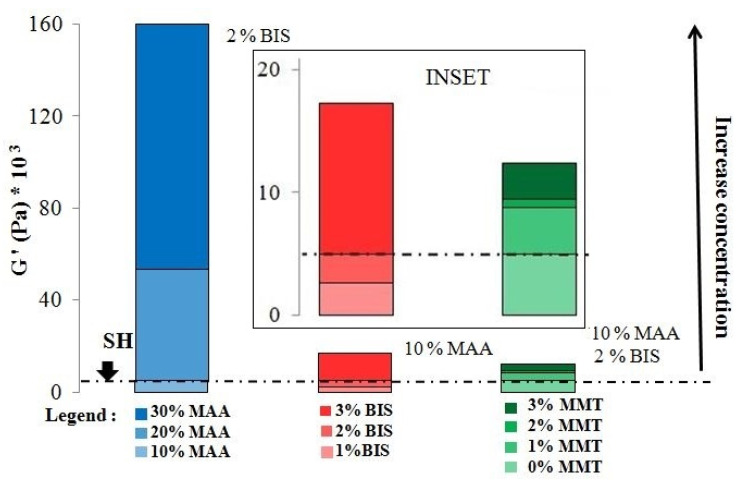
Comparative influence of the monomer, crosslinking agent, and reinforcing agent concentrations on the viscoelastic properties of PMAA hydrogels.

The present paper comparatively discusses, for the first time, the influence of the monomer (MAA), crosslinking agent (BIS), and reinforcing agent (MMT) concentrations employed within the usual range, upon the viscoelastic properties determined on the as-prepared hydrogels, and the water absorption/swelling degree at various pH values of the PMAA hydrogels. It should be mentioned that the two properties investigated here are essential in establishing the applications of hydrogels. The results showed that the most important effect on the viscoelastic properties was displayed by monomer concentration, the tripling of which led to an approximate 30-fold increase in G′ as compared to an increase of only five times in the case of BIS and 1.5 times when the MMT concentration was raised from 1 wt% to 3 wt% (Figure 10).

The swelling studies confirmed, as expected, the pH-sensitive character of the studied hydrogels, the ESD increasing with the pH of the medium in all cases. The increase in the concentrations of the monomer and crosslinking agent led to a decrease in water absorption due to the increase in the crosslinking density of the hydrogels in both cases.

The amplitude of the effect of the monomer and BIS concentrations on water absorption was similar at pH 5.4 and 7.4, but they behaved very differently at acidic pH, when the effect of the monomer amount was much stronger. Further, the pH increase had different effects in the case of these two hydrogels series. Namely, while in the case of hydrogels obtained at different monomer concentrations, the (ESD at pH 7.4)/(ESD at pH 1.2) ratio practically did not depend on the amount of the monomer, the same ratio decreased with the crosslinking agent concentration.

As opposed to the effects of the monomer and crosslinking agent concentrations, the increase in the MMT concentration in the hydrogel resulted in an increase in the swelling degree of the hydrogel at pH 5.4 and 7.4, due to the basic character of the clay, while at pH 1.2, a slight decrease in water absorption was noticed, ascribable to a slight crosslinking effect of the MMT, in agreement with the rheological measurements.

This study is very important due to the use of such hydrogels (pH sensitive) in the medical field as devices for controlled-release drugs and also in the environmental protection and sustainable development field for wastewater purification.

## Figures and Tables

**Table 1:** The composition of the hydrogels investigated.

Sample Code	H_2_O (g) ^1^	MAA (g) ^1^	MAA (wt%) ^2^	BIS (g) ^1^	BIS (mole%) ^3^	APS (g) ^1^	APS (mole%) ^3^	MMT (g) ^1^	MMT wt% ^4^
*SH ^5^*	89.4	10	***10***	0.36	2	0.26	*1*	0	0
H20%MAA	78.8	**20**	20	0.72	2	0.52	1	0	0
H30%MAA	48.16	**30**	30	1.08	2	0.76	1	0	0
H1%BIS	89.6	10	10	0.18	**1**	0.26	1	0	0
H3%BIS	89.2	10	10	0.54	**3**	0.26	1	0	0
H1%MMT	88.4	10	10	0.36	2	0.26	1	**1**	9.65
H2%MMT	87.4	10	10	0.36	2	0.26	1	**2**	19.3
H3%MMT	86.4	10	10	0.36	2	0.26	1	**3**	28.95

^1^ For 100 g amount reaction mixture; ^2^ Based on the whole reaction mass; ^3^ With respect to MAA; ^4^ With respect to the total monomer concentration; ^5^ Standard hydrogel (the hydrogel with the standard composition: 10%MAA-2%BIS-0%MMT).

**Table 2:** Dependence of the viscoelastic moduli (frequency sweep measurements) and some structural network parameters on the monomer concentration **^a^**.

Sample Code	G′ (Pa) ^b^	G″ (Pa) ^b^	${\bar{M}}_{c}\;(\mathrm{Da}){\;}^{c}$	$\rho_{c}\times 10^{-4}\;(\mathrm{mol}/{\mathrm{cm}}^{3}){\;}^{d}$
SH ^e^	5.05 × 10^3^	0.92 × 10^3^	874.9	14.7
H20% MAA	53.8 × 10^3^	13.3 × 10^3^	368.7	35.0
H30% MAA	160 × 10^3^	46 × 10^3^	193.8	66.6

^a^ Hydrogel composition: MAA, 2 mol% BIS based on MAA, 0% MMT; ^b^ G′ and G″ at 1 Hz obtained during the frequency sweep analyses; ^c^ Average molecular weight between crosslinks at pH 1.2, calculated with Equation (3); ^d^ Crosslinking density at pH 1.2, calculated with Equation (5); ^e^ SH: 10%MAA, 2 mol%BIS based on MAA, 0%MMT.

**Table 3:** Dependence of the viscoelastic moduli (frequency sweep measurements) and some structural network parameters on the crosslinking agent concentration ^a^.

Sample Code	G′ (Pa) ^b^	G″ (Pa) ^b^	${\bar{M}}_{c}\;(\mathrm{Da}){\;}^{c}$	$\rho_{c}\times 10^{-4}\;(\mathrm{mol}/{\mathrm{cm}}^{3}){\;}^{d}$
H1%BIS	2.63 × 10^3^	0.31 × 10^3^	915.2	14.1
SH ^e^	5.05 × 10^3^	0.92 × 10^3^	874.9	14.7
H3% BIS	17.3 × 10^3^	5.37 × 10^3^	595.4	21.7

^a^ Hydrogel composition: BIS, 10% MAA based on the whole hydrogel composition, 0% MMT; ^b^ G′ and G″ at 1 Hz obtained during the frequency sweep analyses; ^c^ Average molecular weight between crosslinks at pH 1.2, calculated with Equation (3); ^d^ Crosslinking density at pH 1.2, calculated with Equation (5); ^e^ SH: 10%MAA, 2 mol%BIS based on MAA, 0%MMT.

**Table 4:** Characteristic FT-IR peak values of the investigated samples ^a^.

Sample Code	PMAA (cm^−1^)	MMT (cm^−1^)
MMT	–	999
SH ^b^	1692; 1158	–
H1%MMT	1693; 1164	1046
H2%MMT	1695; 1167	1042
H3%MMT	1693; 1168	1039

^a^ Hydrogel composition: MMT, 10% MAA based on the whole hydrogel composition, 2 mol%BIS based on MAA; ^b^ SH: 10%MAA, 2 mol%BIS based on MAA, 0%MMT.

**Table 5:** The XRD values obtained for the investigated xerogels ^a^.

Sample Code	2 Theta (Å)	d-Spacing (nm)
MMT	6.8	13
H1%MMT	6.63	13.3
H2%MMT	6.5	13.5
H3%MMT	6.7	13.2

^a^ Xerogel composition: MMT, 10% MAA based on the whole hydrogel composition, 2 mol%BIS based on MAA.

**Table 6:** The TGA/DTG results obtained for investigated composite hydrogels ^a^.

Sample Code	Weight Loss Intervals (%)			Decomposition Temperatures (°C)		Residue (%) at 700 °C
	0–120 °C	120–300 °C	300–700 °C	T_1_	T_2_	
SH ^b^	2.87	18.96	71.9	225	424.5	6.31
H1%MMT	3.22	18.14	66.35	222.2	411.6	12.27
H2%MMT	3.44	17.24	59.40	221.3	401.9	19.90
H3%MMT	3.173	16.44	55.16	220.5	393.2	25.26

^a^ Hydrogel composition: MMT, 10% MAA based on the whole hydrogel composition, 2 mol%BIS based on MAA. ^b^ SH: 10%MAA, 2 mol%BIS based on MAA, 0%MMT.

**Table 7:** Dependence of the viscoelastic moduli (frequency sweep measurements) and some structural network parameters on MMT concentration ^a^.

Sample Code	G′ (Pa) ^b^	G″ (Pa) ^b^	${\bar{M}}_{c}\;(\mathrm{Da}){\;}^{c}$	$\rho_{c}\times 10^{-4}\;(\mathrm{mol}/{\mathrm{cm}}^{3}){\;}^{d}$
SH ^e^	5.05 × 10^3^	0.93 × 10^3^	874.9	14.7
H1%MMT	8.8 × 10^3^	1.67 × 10^3^	904.3	14.3
H2%MMT	9.5 × 10^3^	1.52 × 10^3^	774.0	16.7
H3%MMT	12.4 × 10^3^	2.04 × 10^3^	689.5	18.7

^a^ Hydrogel composition: MMT, 10% MAA based on the whole hydrogel composition, 2 mol%BIS based on MAA; ^b^ G′ and G″ at 1 Hz obtained during the frequency sweep analyses; ^c^ Average molecular weight between crosslinks at pH 1.2, calculated with Equation (3); ^d^ Crosslinking density at pH 1.2, calculated with Equation (5); ^e^ SH: 10%MAA, 2 mol%BIS based on MAA, 0%MMT.

## Data Availability

Data sharing not available.

## Supplementary Materials

The following are available online at https://www.mdpi.com/article/10.3390/ma14092305/s1, Figure S1: The appearance of hy-drogels at different pH values: (a) SH sample (10%MAA-2%BIS-0%MMT); (b) H1%BIS sample (10%MAA-1%BIS-0%MMT); (c) H2%MMT (10%MAA-2%BIS-2%MMT). Figure S2: Mechanism of the swelling process. Figure S3: SEM micrographs of lyophilized H2%MMT samples swollen at different pH values: (a) pH 1.2; (b) pH 5.4; (c) pH 7.4.
