# Supplementary material for: The Effects of Monomer, Crosslinking Agent, and Filler Concentrations on the Viscoelastic and Swelling Properties of Poly(methacrylic acid) Hydrogels: A Comparison
Source: Materials (Basel). 2021 Apr 29;14(9):2305. doi: 10.3390/ma14092305 (PMC8124361; doi:10.3390/ma14092305)
Supplement: Supplementary file 1 [file materials-14-02305-s001.zip › materials-1158908-supplementary.pdf]

materials

Supplementary Material

The Effects of Monomer, Crosslinking Agent, and Filler Concentrations on the Viscoelastic and Swelling Properties of poly(methacrylic acid) Hydrogels: A Comparison

Claudia Mihaela Ninciuleanu [1,2], Raluca Ianchis [1], Elvira Alexandrescu [1], Catalin Ionut Mihaescu [1], Cristina Scomoroscenco [1], Cristina Lavinia Nistor [1], Silviu Preda [3], Cristian Petcu [1,*] and Mircea Teodorescu [2,*]

[1] National Institute for Research and Development in Chemistry and Petrochemistry-ICECHIM, Spl. Independentei 202, 060021 Bucharest, Romania; claudia.ninciuleanu@yahoo.com (C.M.N.); ralumoc@yahoo.com (R.I.); elviraalexandrescu@yahoo.com (E.A.); mihaescu_catalin96@yahoo.com (C.I.M.); scomoroscencocristina@gmail.com (C.S.); lc_nistor@yahoo.com (C.L.N.)

[2] Department of Bioresources and Polymer Science, Faculty of Applied Chemistry and Materials Science, Politehnica University of Bucharest, 1-7 Gh. Polizu Street, 011061 Bucharest, Romania

[3] Institute of Physical Chemistry "Ilie Murgulescu", Romanian Academy, Spl. Independentei 202, 6th district, P.O. Box 194, 060021 Bucharest, Romania; predas01@yahoo.co.uk

* Correspondence: cpetcu@icf.ro (C.P.); mircea.teodorescu@upb.ro (M.T.)

Citation: Ninciuleanu, C.M.; Ianchis, R.; Alexandrescu, E.; Mihaescu, C.I.; Scomoroscenco, C.; Nistor, C.L.; Preda, S.; Petcu, C.; Teodorescu, M. The Effects of Monomer, Crosslinking Agent and Filler Concentrations upon the Viscoelastic and Swelling Properties of poly(methacrylic acid) Hydrogels: A Comparison. *Materials* **2021**, *14*, 2305. https://doi.org/10.3390/ma14092305

Academic Editor: Isabel Izquierdo Barba

Received: 10 March 2021
Accepted: 27 April 2021
Published: 29 April 2021

Publisher's Note: MDPI stays neutral with regard to jurisdictional claims in published maps and institutional affiliations.

Copyright: © 2021 by the authors. Licensee MDPI, Basel, Switzerland. This article is an open access article distributed under the terms and conditions of the Creative Commons Attribution (CC BY) license (http://creativecommons.org/licenses/by/4.0/).

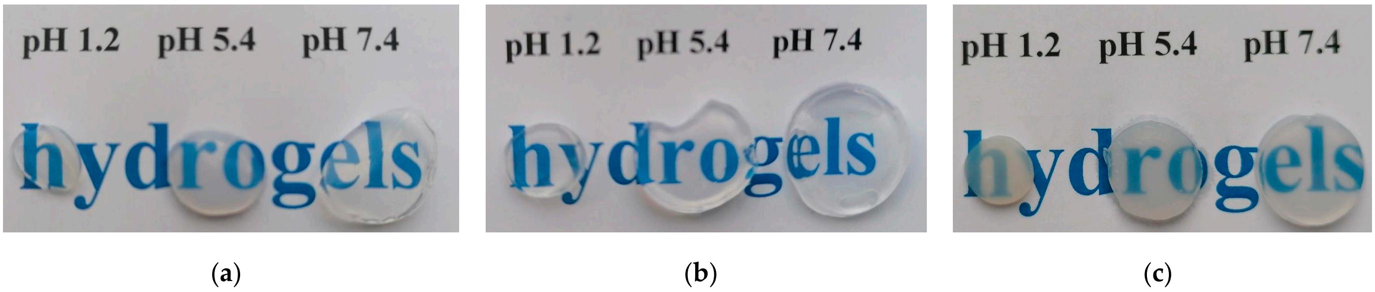

Figure S1. The appearance of hydrogels at different pH values: (**a**) **SH sample** (10%MAA-2%BIS-0%MMT); (**b**) **H1%BIS** sample (10%MAA-1%BIS-0%MMT); (**c**) **H2%MMT** (10%MAA-2%BIS-2%MMT).

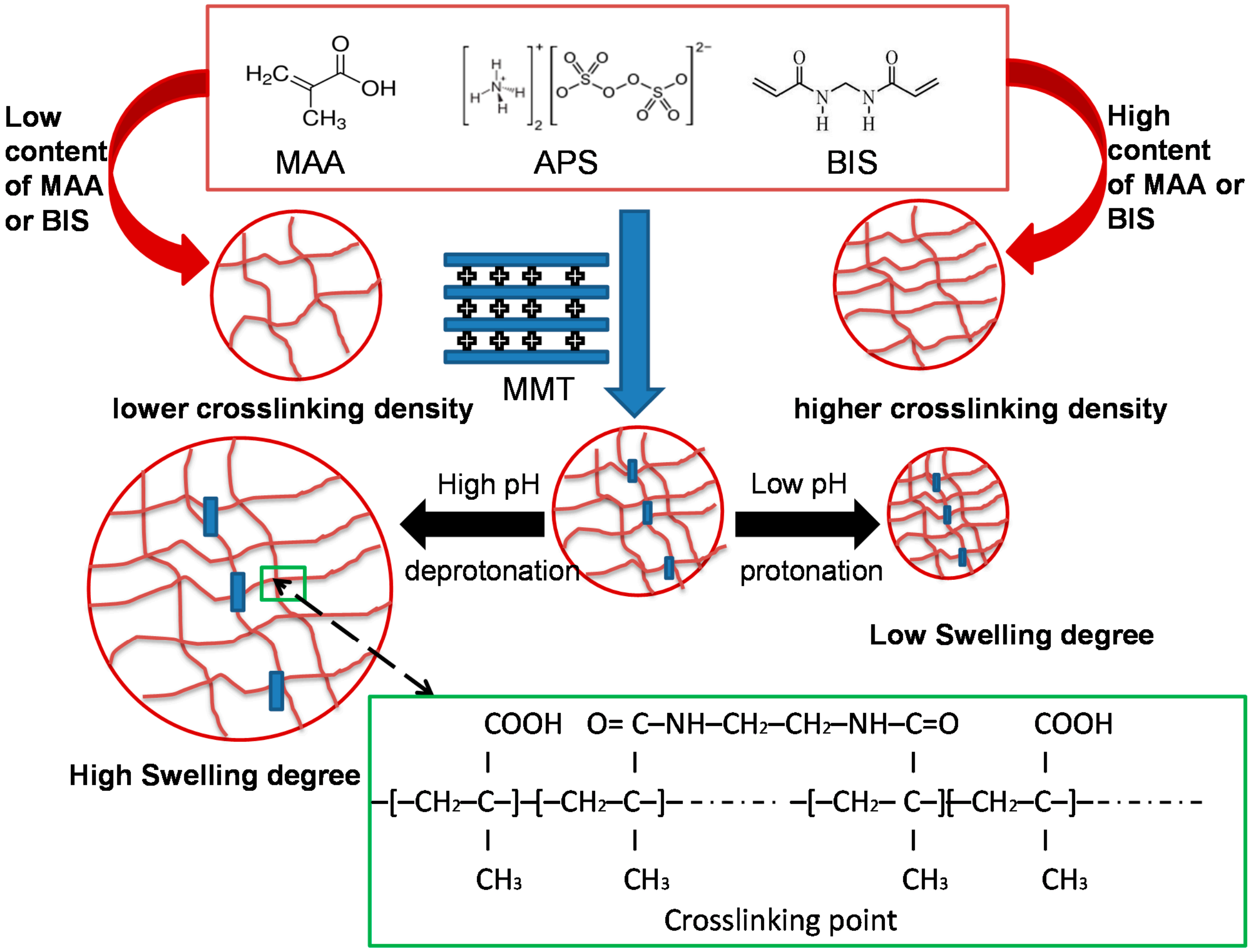

Figure S2. Mechanism of the swelling process.

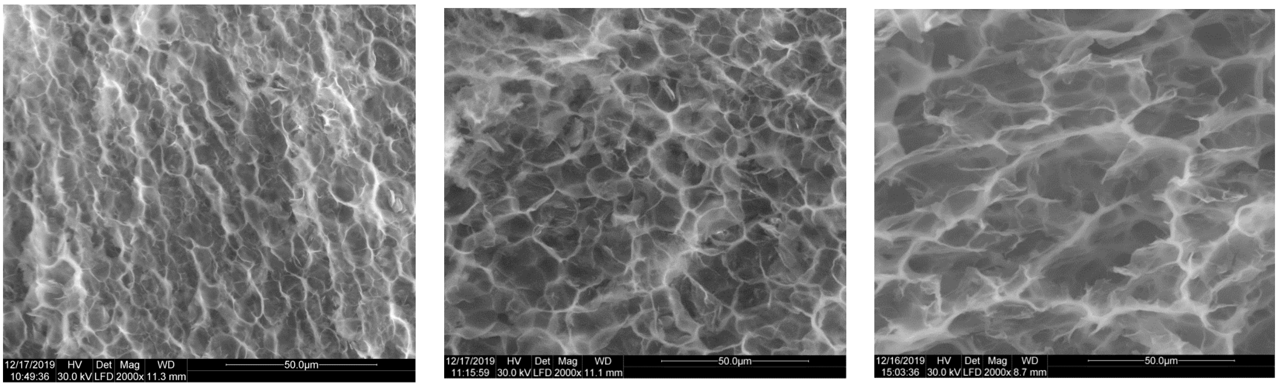

(**a**) (**b**) (**c**)

Figure S3. SEM micrographs of lyophilized **H2%MMT samples** swollen at different pH values: (**a**) pH 1.2; (**b**) pH 5.4; (**c**) pH 7.4.
